# Loss of heterozygosity on chromosome 18q is associated with muscle-invasive transitional cell carcinoma of the bladder.

**DOI:** 10.1038/bjc.1994.376

**Published:** 1994-10

**Authors:** S. F. Brewster, J. C. Gingell, S. Browne, K. W. Brown

**Affiliations:** Department of Urology, Southmead Hospital, Bristol, UK.

## Abstract

**Images:**


Br. J. Cancer (1994), 70, 697-700 © Macmillan Press Ltd., 1994

### Loss of heterozygosity on chromosome 18q is associated with muscle-invasive transitional cell carcinoma of the bladder

S.F. Brewster[1,2], J.C. Gingell[1], S. Browne[2] & K.W. Brown[2]

[1]Department of Urology, Southmead Hospital, Bristol BS10 5NB, UK; [2]Department of Pathology & Microbiology, School of Medical Sciences, University of Bristol, BS8 1TD, UK.

**Summary** Somatic allelic loss is regarded as a hallmark of tumour-suppressor gene (TSG) inactivation. Thirty-one human bladder transitional cell carcinomas (TCCs) were examined for allelic loss at five chromosome 18q loci, including the DCC gene (deleted in colorectal carcinoma) and at chromosome 11p15 in a restriction fragment length polymorphism analysis. Allelic loss was observed at one or more 18q loci in 9/26 (35%) samples, associated with muscle-invasive disease ($P<0.02$). Allelic loss was observed at DCC in 8/24 (33%) samples, associated with muscle-invasive disease ($P=0.05$). Three out of the five evaluable *recurrent* TCCs exhibited allelic loss at DCC, two of which were superficial. No allelic losses were detected at other 18q loci in tumours which retained both DCC alleles. Allelic loss was observed at 11p15 in 5/20 (25%) tumours. These data suggest the presence of a late-acting TSG located on 18q in TCC bladder cancer. DCC is a candidate gene since it lies within the region of most common deletion (18q21.3-qter).

Multistage epithelial carcinogenesis is considered to result from an accumulation of somatic genetic abnormalities including the inactivation of tumour-suppressor genes (TSG) and the activation of cellular proto-oncogenes. TSGs are a heterogeneous group of genes whose inactivation may result in deregulated growth and clonal expansion or the acquisition of invasive or metastatic potential (Brewster *et al.*, 1992). In clinical oncology, TSGs or their products may be exploited as screening tools for inherited cancers, as prognostic indicators and potentially as targets for corrective gene therapy (Brewster & Simons, 1994).

Non-random loss of chromosomal regions occurs frequently in neoplasia (Mitelman *et al.*, 1991). Although loss of chromosome regions of less than 25% of tumour samples may be considered random and of questionable significance (Seizinger *et al.*, 1991), clonal somatic allelic loss is considered to be a hallmark of TSG inactivation since the loss can unmask a 'recessive' mutant allele (Cavenee *et al.*, 1983). Somatic allelic loss may be demonstrated as loss of heterozygosity (LOH) by restriction fragment length polymorphism (RFLP) analysis of paired constitutional and tumour DNA.

The DCC gene (deleted in colorectal carcinoma) (Fearon *et al.*, 1990) maps to chromosome 18q21.3, spanning >3 Mb to include at least 29 exons. The predicted DCC protein shows sequence homology with neural cell adhesion molecules and other cell-surface glycoproteins; it is suggested that DCC plays a role in maintaining cell-cell interaction. 18q LOH occurs in over 70% of colorectal carcinomas, 47% of advanced adenomas and less than 15% of earlier adenomas (Vogelstein *et al.*, 1988). In addition, LOH at DCC has been observed in 61% of gastric cancers (Uchino *et al.*, 1992), 31% of breast cancers (Thompson *et al.*, 1993), 44% of ovarian cancers (Chenevix-Trench *et al.*, 1992) and 26% of prostate cancers (Brewster *et al.*, 1994). Deletion mapping data from the last two studies have suggested the presence of a second TSG located telomeric to DCC on chromosome 18q.

Bladder cancer caused 5,659 deaths in the UK in 1992, ranking fifth (males) and 11th (females) in terms of cancer-related mortality (CRC Education Department, personal communication). Over 95% of all bladder cancers in industrialised countries are transitional cell carcinomas (TCCs). RFLP studies of TCC have shown frequent (>50%) LOH on chromosome 9q (Tsai *et al.*, 1990; Cairns *et al.*, 1993). It has been proposed that an early-acting TSG resides on 9q, since LOH is often the only genetic abnormality observed in superficial papillary TCC. In invasive TCC, LOH has been observed at 11p15 in 33-42% samples (Fearon *et al.*, 1985; Tsai *et al.*, 1990; Presti *et al.*, 1991), but similarly a TSG remains to be identified here. LOH within the retinoblastoma susceptibility gene (*Rb1*) gene (13q14) was observed in 56% of muscle-invasive samples (Cairns *et al.*, 1992), on 17p in 50-81% of muscle-invasive samples (Tsai *et al.*, 1990; Habuchi *et al.*, 1993) and at a locus (18q22) distal to DCC in 4/12 (33%) muscle-invasive samples, but in 0/5 superficial tumours (Presti *et al.*, 1991). Dalbagni *et al.* (1993) reported LOH on 18q in 26% of TCCs. Reznikoff *et al.* (1993) have developed a system in which transformation and other neoplastic characteristics of cultured immortalised human urothelial cells may be studied by exposing the cells to carcinogens such as 4-aminobiphenyl. Among other changes, 18q deletions were observed by RFLP analysis of derivative tumours in nude mice.

Here we present the results of an RFLP analysis in which DNA from a series of TCCs was studied for allelic loss at five chromosome 18q loci including DCC (Figure 1), aiming to define a region of common deletion and, furthermore, its relationship to DCC. In order to provide a comparison between our sample population and those of previous studies, we chose to examine our series for allelic loss at chromosome 11p15.

### Materials and methods

### *Tissues*

Thirty-one TCCs were included in the analysis, consecutively obtained from 20 men and 11 women. Ten were recurrent tumours. Samples were transected longitudinally, half snap frozen and stored at −70°C and half submitted for histological examination. Those samples containing less than 70% malignant cells were excluded. Tumours were graded histologically as G1-3 (World Health Organization classification) and pathologically staged by the TNM system (Hunter, 1978). Herein, pTa indicates non-invasive superficial papillary tumour; pT1 indicates invasion of the lamina propria; and pT2-3 indicates muscle-invasive tumour. Additionally, 5 ml of heparinised venous blood was obtained from each patient.

Correspondence: S.F. Brewster, Bristol Urological Institute, Southmead Hospital, Bristol BS10 5NB, UK.
Received 21 December 1993; and in revised form 5 April 1994.

*DNA extraction and Southern blot hybridisation analysis*

High molecular weight tumour and lymphocyte DNA was extracted as described previously (Brewster *et al.*, 1994). Paired DNA samples were digested with *Msp*I, *Eco*RI, *Bgl*II, *Pst*I or *Hinf*I and electrohoresed on 1% agarose gels. Southern blots (Hybond N+, Amersham, UK) were hybridised to random-primer $^{32}P$-labelled DNA probes (Table I) in rapid hybridisation buffer (Amersham) and washed to a final stringency of either 2 × SSC/0.5% SDS or 0.5 × SSC/0.1% SDS at 65°C. Optical scanning densitometry of autoradiographs was undertaken using a Biorad videodensitometer in certain cases to investigate visual impressions of allelic loss. LOH was considered present when one of two polymorphic alleles present in lymphocyte DNA was reduced in the tumour DNA by at least 50%, so making allowance for genetic heterogeneity and contaminating normal DNA in tumour samples. Samples showing LOH were subjected to DNA 'fingerprinting' using a 720 bp minisatellite probe (Jeffreys *et al.*, 1985).

**Figure 1** A schematic diagram of chromosome 18, showing the relationship of DCC to the four anonymous 18q RFLP markers used.

**Table I** DNA probes used to detect RFLPs

| *Probe name* | *Locus/gene* | *Restriction enzyme* | *Reference* |
|---|---|---|---|
| pL2.7 | 18q12.2 | *Pst*I | Hofker *et al.* (1986) |
| OL VII A8 | 18q12.1-21.1 | *Msp*I | Delattre *et al.* (1987) |
| JOSH 4.4 | 18q21.3 DCC | *Pst*I | Simons *et al.* (1992) |
| SAM 1.1 | 18q21.3/DCC | *Eco*RI | Simons *et al.* (1992) |
| p15-65 | 18q21.3 DCC | *Msp*I | Fearon *et al.* (1990) |
| pOS-4 | 18q22 | *Pst*I/*Taq*I | Nishisho *et al.* (1987) |
| pL159-1 | 18q23 | *Pst*I | Kazazian *et al.* (1986) |
| p2.1 | 11p15 | *Pst*I/*Hinf*I | Brookes *et al.* (1989) |
| pEJ6.6 | 11p15.5 | *Msp*I | Krontiris *et al.* (1993) |

*Statistical analysis*

The Fisher-Irwin exact chi-square ($\chi^2$) test applying Yates' correction was used for statistical analysis of the results.

### Results

Results for allelic status on chromosome 18q are summarised in Table II. DNA fingerprinting confirmed tumour/blood identity in all but one sample exhibiting LOH; in this case (tumour 5) insufficient DNA was available.

*Chromosome 18q and DCC*

In total, 26/31 (84%) tumours were evaluable at one or more chromosome 18q locis, of which nine (35%) exhibited LOH, correlating significantly with advanced pathological stage: only 4/20 superficial (pTa/pT1) tumours exhibited 18q allele loss compared with 5/6 muscle-invasive (pT2/3) tumours ($\chi^2 = 5.62$, $P = 0.018$).

Combining three RFLP markers for DCC, 24 (77%) samples were informative. LOH at DCC was observed in eight (33%) tumours, examples of which are shown in Figure 2. Five recurrent TCCs were informative at DCC, of which three showed LOH; two were pTa and the other was pT2/3. LOH at DCC was significantly associated with muscle invasion, observed in 4/19 pTa/pT1 and 4/5 pT2/3 samples ($\chi^2 = 3.82$, $P = 0.05$), and muscle invasion or recurrence ($\chi^2 = 4.2$, $P = 0.04$), but non-significantly with *all* (pT1 + pT2/3) invasive disease ($P = 0.3$), grade 3 disease ($P = 0.6$) or recurrent disease alone ($P = 0.6$).

Four out of eight tumours showing LOH at DCC exhibited LOH at one of the two centromeric loci; two tumours, 18 and 26, retained heterozygosity with at least one of these markers. All evaluable tumours exhibiting LOH at DCC also exhibited LOH telomeric to DCC. Tumour 21 was evaluable at only one locus, centromeric to DCC, which showed LOH. No tumour retaining heterozygosity at DCC

**Table II** Chromosome 18q allelic status in 31 TCCs

| *Tumour: grade/stage* | | *18q status* |
|---|---|---|
| 1 | G2pTa | No LOH |
| 2 | G2pTa | No LOH |
| 3 | G2pTa R | No LOH |
| 4 | G2pTa R | NI |
| 5 | G2pTa | LOH |
| 6 | G2pTa | No LOH |
| 7 | G2pTa | No LOH |
| 8 | G1pTa | No LOH |
| 9 | G1pTa | No LOH |
| 10 | G2pTa R | NI |
| 11 | G2pTa R | NI |
| 12 | G2pTa R | No LOH |
| 13 | G2pTa | No LOH |
| 14 | G2pTa | No LOH |
| 15 | G2pTa R | LOH |
| 16 | G1pTa | No LOH |
| 17 | G2pTa R | LOH |
| 18 | G2pT1 | LOH |
| 19 | G3pT2/3 R | NI |
| 20 | G3pT2/3 | LOH |
| 21 | G3pT2/3 | LOH |
| 22 | G2pT2/3 | LOH |
| 23 | G3pT1 | No LOH |
| 24 | G2pT1 | No LOH |
| 25 | G2pT2/3 R | NI |
| 26 | G3pT2/3 | LOH |
| 27 | G3pT1 | No LOH |
| 28 | G3pT2/3 | NI |
| 29 | G2pT2/3 | No LOH |
| 30 | G3pT2/3 | LOH |
| 31 | G3pT1 | No LOH |

LOH, loss of heterozygosity; NI, not informative; R, recurrent tumour.

showed LOH elsewhere on 18q; the region of commonest deletion in this series was therefore 18q21.3-qter (Table III).

### *Chromosome 11p*

Twenty out of 24 (82%) tumours were informative with one of the two 11p15 markers, of which five (25%) exhibited LOH (Figure 3). This alteration was observed in 1/9 (11%) pTa and 4/11 (36%) invasive (pT1-3) tumours; this was not statistically significant ($\chi^2 = 0.61$, $P = 0.44$). There were no statistically significant associations between 11p15 LOH and muscle-invasive, recurrent or G3 disease ($P = 0.42$, $P1$, $P = 1$ respectively).

**Figure 2** Autoradiographs showing LOH at DCC (**a**) in tumour 22, (**b**) in tumour 15 and (**c**) in tumour 18. B = blood, T = tumour, A = allele.

**Figure 3** Autoradiograph showing LOH at 11p15 in tumour 31. B = blood, T = tumour, A = allele.

### Discussion

The theory of multistep carcinogenesis (Nordling, 1953) was given a molecular basis by the demonstration of an accumulation of genetic events resulting in the activation of oncogenes and the loss of TSGs in colorectal tumours of increasing grade and stage (Fearon & Vogelstein, 1990). There is mounting evidence that the clinicopathological course of bladder cancer is governed by such an accumulation. The sequence of events appears to be initially associated with loss of genetic material on chromosome 9q; the invasive phenotype is associated with losses on 11p and subsequently on 3p, 13q, 17p and 18q, though many of the pivotal genes, including those on 11p and 18q, remain unidentified. Combinations of these alterations may confer upon tumours the various behavioural phenotypes which characterise the clinical heterogeneity of invasive bladder cancer (Prout *et al.*, 1979).

Multifocality and recurrence are common clinical features of TCC: 70% of G1 and 80-90% of G3 superficial tumours recur after first resection, of which 10-15% will have become invasive. Two theories on the pathogenesis of these phenomena have been proposed. Traditionally, it was held that an inherent or environmental factor rendered the entire urothelium unstable, from renal calyces to prostatic urethra. This 'field change' may explain why patients develop upper urinary tract TCC many years after bladder TCC has been diagnosed and treated. Alternatively, TCC can be viewed as a monoclonal disease with a great propensity to seed. X chromosome inactivation and other somatic allelic changes within 13 TCCs from four patients support this theory (Sidransky *et al.*, 1992). All tumours belonging to the same patient exhibited identical X chromosome inactivation, while normal surrounding transitional cells exhibited random X inactivation. In addition, each evaluable tumour from a given patient exhibited loss of identical 9q alleles but variable 17p and 18q alleles, suggesting that the latter changes occur later during the independent evolution of individual 'multifocal' tumours. Recurrent tumours may also appear by this seeding mechanism.

In the present study, allelic loss on chromosome 18q was

**Table III** Chromosome 18q allelic deletion mapping

| *Probe (total informative)* | *pL2.7 (8) 18q12.2* | *OLVII A8 (18) 18q12.1-21.1* | *SAM 1.1 (15) DCC: 18q21.3* | *JOSH 4.4 (19) DCC: 18q21.3* | *p15-65 (16) DCC: 18q21.3* | *pOS-4 (9) 18q22* | *pL 159-1 (11) 18q23* |
|---|---|---|---|---|---|---|---|
| Tumour: grade, stage | | | | | | | |
| 5 G2pTa | NI | LOH | LOH | LOH | NI | NI | LOH |
| 15 G2pTa R | NI | LOH | LOH | LOH | NI | NI | LOH |
| 17 G2pTa R | NI | LOH | LOH | LOH | LOH | LOH | NI |
| 18 G2pT1 | No LOH | No LOH | - | LOH | NI | LOH | NI |
| 20 G2pTa R | LOH | NI | - | LOH | LOH | - | LOH |
| 21 G3pT2/3 | NI | LOH | NI | NI | NI | NI | NI |
| 22 G2pT2/3 R | NI | NI | LOH | LOH | LOH | LOH | LOH |
| 26 G3pT2/3 | NI | No LOH | LOH | LOH | NI | LOH | NI |
| 30 G3pT2/3 | LOH | NI | LOH | LOH | LOH | NI | NI |

LOH, loss of heterozygosity; NI, not informative; R, recurrent tumour.

observed in one-third of informative TCCs, significantly associated with muscle invasion. This is slightly (but not significantly) more frequent than previously reported (Presti et al., 1991; Dalbagni et al., 1993). Two explanations are offered. First, only the anonymous probe pOS-4, mapping telomeric to DCC, was used in previous studies. Second, it is not clear from either previous study whether any non-invasive TCC samples were recurrent. Two of the three pTa TCCs exhibiting 18q LOH in the present study were recurrent and, as a group, recurrent and or muscle-invasive disease was significantly associated with this event. DCC was included in the deleted regions of *all* the tumours exhibiting allelic loss on 18q. The region of commonest deletion was 18q21.3-qter; no interstitial 18q deletions were observed. One-quarter of TCCs studied exhibited loss of 11p15 alleles. This change was observed in tumours of all stages, increasing with grade and stage at a slightly (but not significantly) lower frequency than previous observations of 11p LOH (see earlier). We conclude from this that our sample tumour population is comparable to those of previous studies.

A suppressor role for DCC in bladder cancer is supported by preliminary studies of 18q-deleted TCC cells in culture, in which DCC expression was undetectable (C.A. Reznikoff, American Society of Basic Urological Research Annual meeting, 1993, and personal communication). Following transfection with DCC cDNA, reversion of malignant phenotype and detectable DCC transcript was observed in these cells. Similar suppression was not observed when these cells were transfected with mutant DCC cDNA under the same conditions.

It is concluded that loss of genetic material on 18q21.3-qter, a region including DCC, is associated with muscle-invasive disease and occurs frequently in recurrent disease. DCC is therefore a potential target for 18q deletion and is thus a candidate TSG in TCC.

The authors wish to thank Professor B. Vogelstein, Johns Hopkins Oncology Centre, Baltimore, USA, from whom the DCC probes were obtained. We are indebted to all the consultants in the Departments of Urology and Histopathology at Southmead Hospital, in particular Dr A. MacIver. This work was supported by a research grant from the South-West Regional Health Authority, England.

### References

BREWSTER, S.F., BROWNE, S. & BROWN, K.W. (1994). Somatic allelic losses at the DCC, p53, *nm23*-H1 and APC tumor suppressor gene loci in prostate cancers. *J. Urol.*, **151**, 1073-1077.

BREWSTER, S.F., GINGELL, J.C. & BROWN, K.W. (1992). Tumour suppressor genes in urinary tract oncology. *Br. J. Urol.*, **70**, 585-590.

BREWSTER, S.F. & SIMONS, J.W. (1994) Gene therapy in urological oncology: principles, strategies and potential. *Eur. J. Urol.*, **25**, 177-182.

BROOKES, A.J., HEDGE, P.H. & SOLOMON, E. (1989). A highly polymorphic locus on chromosome 11 which has homology to a collagen triple-helix coding sequence. *Nucleic Acids Res.*, **17**, 1792.

CAIRNS, P., PROCTOR, A.J. & KNOWLES, M.A. (1992). Loss of heterozygosity at the RB locus is frequent and correlates with muscle invasion in bladder carcinoma. *Oncogene*, **6**, 2305-2309.

CAIRNS, P., SHAW, M.E. & KNOWLES, M.A. (1993). Initiation of bladder cancer may involve deletion of a tumour-suppressor gene on chromosome 9. *Oncogene*, **8**, 1083-1085.

CAVENEE, W.K., DRYJA, T.P. & PHILLIPS, R.A. (1983). Expression of recessive alleles by chromosomal mechanisms in retinoblastoma. *Nature*, **305**, 775-784.

CHENEVIX-TRENCH, G., LEARY, J., KERR, J., MICHEL, J., KEFFORD, R., HURST, T., PARSONS, P.G., FREIDLANDER, M. & KHOO, S.K. (1992). Frequent loss of heterozygosity on chromosome 18 in ovarian adenocarcinoma which does not always include the DCC locus. *Oncogene*, **7**, 1059-1065.

DALBAGNI, G., PRESTI, J.C., REUTER, V.E., FAIR, W.R. & CORDON-CARDO, C. (1993). Genetic alterations in bladder cancer. *Lancet*, **342**, 469-471.

DELATTRE, O., BERNARD, A., MARLHENS, F., MONPEZAT, J., DUTRILLAUX, B. THOMAS, G. (1987). RFLP identified by the anonymous DNA segment OL VIII A8 at 18q11 (HGM no. D18S7). *Nucleic Acids Res.*, **15**, 1343.

FEARON, E.R. & VOGELSTEIN, B. (1990). A genetic model for colorectal tumorigenesis. *Cell*, **61**, 759-767.

FEARON, E.R., FEINBERG, A.P., HAMILTON, S.H. & VOGELSTEIN, B. (1985). Loss of genes on the short arm of chromosome 11 in bladder cancer. *Nature*, **318**, 377-380.

FEARON, E.R., CHO, K.R., NIGRO, J.M., KERN, S.E. & VOGELSTEIN, B. (1990). Identification of a chromosome 18q gene that is altered in colorectal cancer. *Science*, **247**, 49-55.

HABUCHI, T., OGAWA, O., KAKEHI, Y., OGURA, K., KOSHIBA, M., HAMAZAKI, S., TAKAHASHI, R., SUGIYAMA, T. & YOSHIDA, O. (1993). Accumulated allelic losses in the development of invasive urothelial cancer. *Int. J. Cancer*, **53**, 579-584.

HOFKER, M.H., SKRAASTAD, M.I., BERGEN, A.A., WAPENAAR, M.C., BAKKER, E., MILLINGTON-WARD, A., VAN OMMEN, G.J. & PEARSON, P.L. (1986). The X chromosome shows less genetic variation at restriction sites than autosomes. *Am. J. Hum. Genet.*, **39**, 438-451.

HUNTER, M.H. (ed.) (1978). TNM *Classification of Malignant Tumours*, 3rd edn. UICC: Geneva.

JEFFREYS, A.J., WILSON, V. & THEIN, S.L. (1985). Hypervariable 'minisatellite' regions in human DNA. *Nature*, **314**, 67-73.

KAZAZIAN, H.H., ORKIN, S.H., BOEHM, C.D., GOFF, S.C., WONG, C., DOWLING, C.E., NEWBURGER, P.E., KNOWLTON, R.G., BROWN, V. & DONIS-KELLER, H. (1986). Characterisation of a spontaneous mutation to a β-thalassaemia allele. *Am. J. Hum. Genet.*, **38**, 860-867.

KRONTIRIS, T.G., DEVLIN, B., KARP, D.D., ROBERT, N.J. & RISCH, N. (1993). An association between the risk of cancer and mutations in the H*ras*1 minisatellite locus. *N. Engl. J. Med.*, **329**, 517-523.

MITELMAN, F., KANEKO, Y. & TRENT, J. (1991). Report of the committee on chromosome changes in neoplasia. *Cytogenet. Cell Genet.*, **58**, 1053-1079.

NISHISHO, I., TATEISHI, H., MOTOMURA, K. & MORI, T. (1987). Assignment of the polymorphic locus of OS-4 (D18S5) DNA segment to chromosome region 18q21.3-qter. *Jpn. J. Hum. Genet.*, **32**, 1-7.

NORDLING, C.O. (1953) A new theory on the cancer-inducing mechanism. *Br. J. Cancer*, **7**, 68-72.

PRESTI, J.C., REUTER, V.E., GALAN, T., FAIR, W.R. & CORDON-CARDO, C. (1991). Molecular genetic alterations in superficial and locally advanced human bladder cancer. *Cancer Res.*, **51**, 5405-5409.

PROUT, G.R., GRIFFIN, P.P. & SHIPLEY, W.U. (1979). Bladder carcinoma as a systemic disease. *Cancer*, **43**, 2532-2539.

REZNIKOFF, C.A., KAO, C., MESSING, E.M., NEWTON, M. & SWAMINATHAN, S. (1993). A molecular genetic model of human bladder carcinogenesis. *Semin. Cancer Biol.*, **4**, 143-152.

SEIZINGER, B.R., KLINGER, H.P., JUNIEN, C., NAKAMURA, Y., LE BEAU, M., CAVENEE, W., EMANUEL, B., PONDER, B., NAYLOR, S., MITELMAN, F., LOUIS, D. & MENON, A. (1991). Report of the committee on chromosome and gene loss in human neoplasia. *Cytogenet. Cell Genet.*, **58**, 1080-1096.

SIDRANSKY, D., FROST, P., VON ESCHENBACH, A. & VOGELSTEIN, B. (1992). Clonal origin of bladder cancer. *N. Engl. J. Med.*, **326**, 737-740.

SIMONS, J.W., OLINER, J.D., CHO, K.R., KINZLER, K.W. & VOGELSTEIN, B. (1992). SAM 1.1 and JOSH 4.4: two RFLPs within the human DCC gene. *Hum. Mol. Genet.*, **5**, 352.

THOMPSON, A.M., MORRIS, R.G., WALLACE, M., WYLLIE, A.H., STEEL, C.M. & CARTER, D.C. (1993). Allele loss from 5q21 (APC MCC) and 18q21 (DCC) and DCC mRNA expression in breast cancer. *Br. J. Cancer*, **68**, 64-68.

TSAI, Y.C., NICHOLS, P.W., HITI, A.L. & JONES, P.A. (1990). Allelic losses of chromosomes 9, 11 and 17 in human bladder cancer. *Cancer Res.*, **50**, 44-47.

UCHINO, S., TSUDA, H., NOGUCHI, M., YOKOTA, J., TERADA, M., SAITO, T., KOBAYASHI, M., SUGIMURA, T. & HIROHASHI, S. (1992). Frequent loss of heterozygosity at the DCC locus in gastric cancer. *Cancer Res.*, **52**, 3099-3101.

VOGELSTEIN, B., FEARON, E.R., HAMILTON, S.R., KERN, S.E. & PREISENGER, A.C. (1988). Genetic alterations during colorectal tumor development. *N. Engl. J. Med.*, **319**, 525-532.


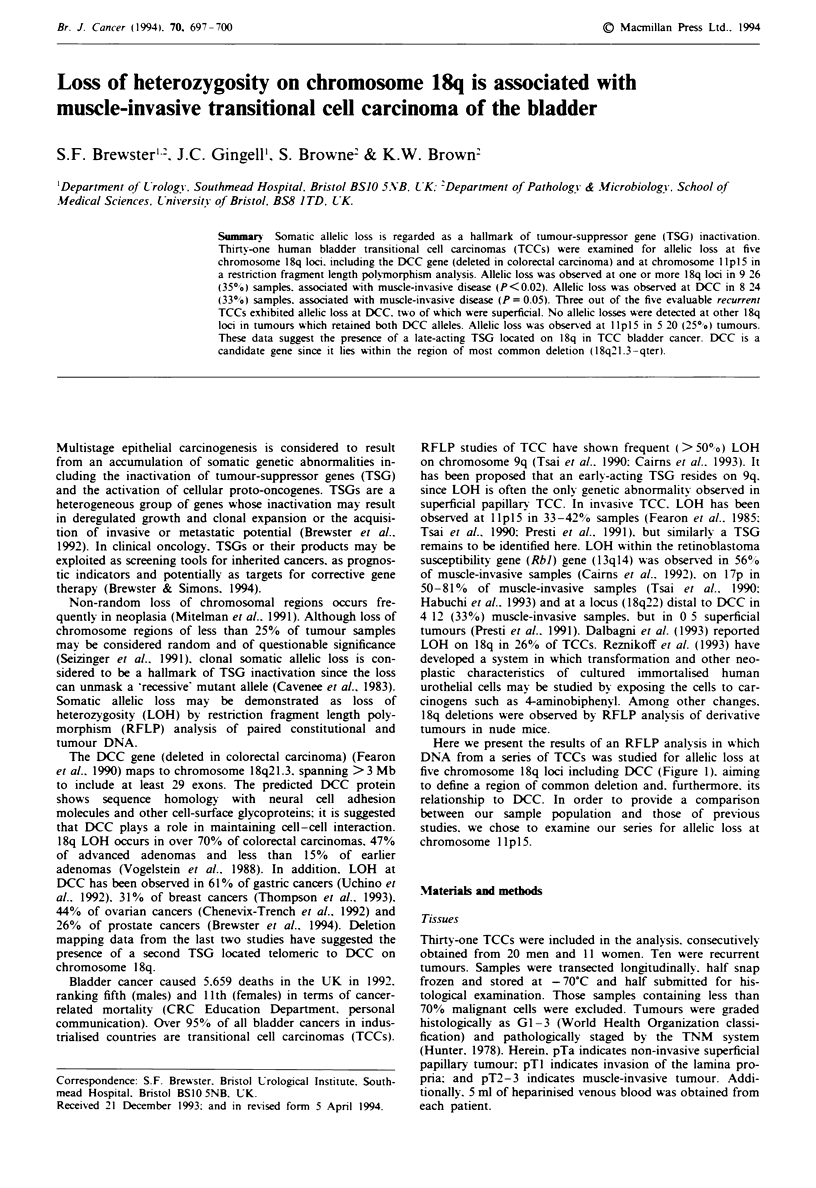

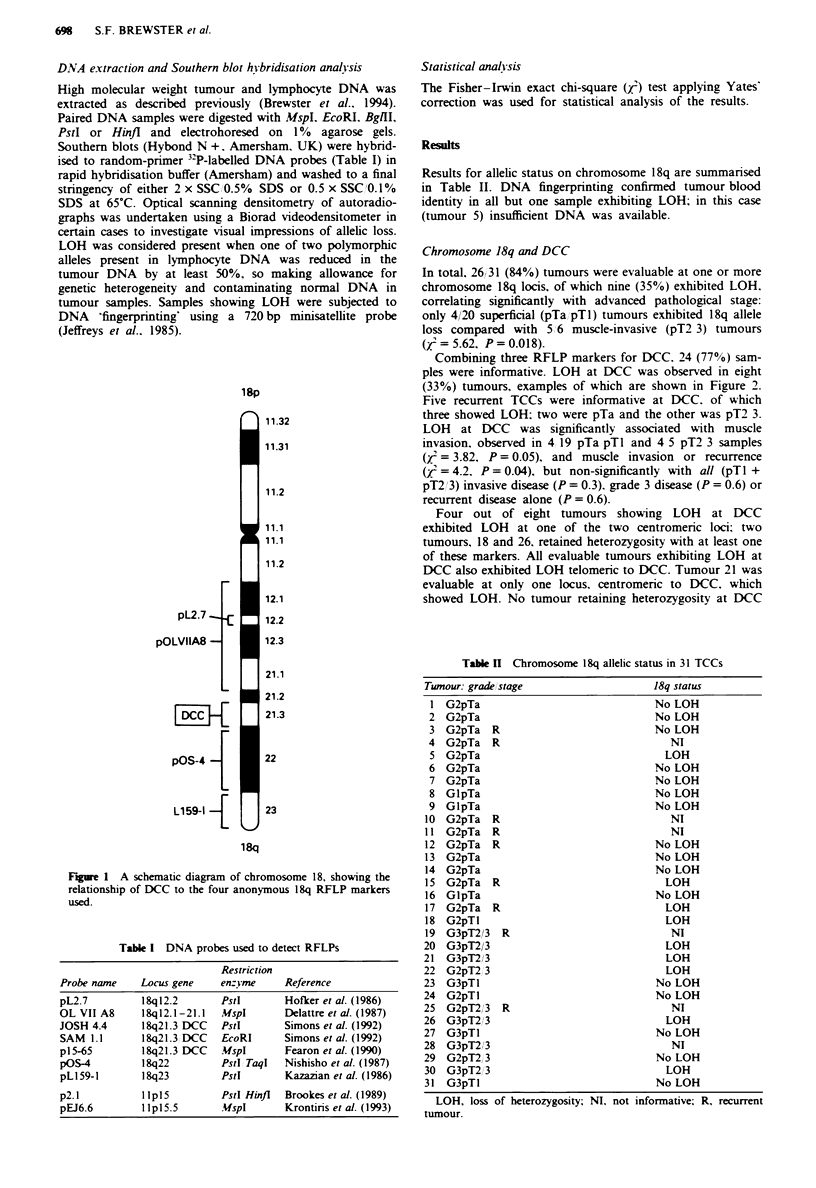

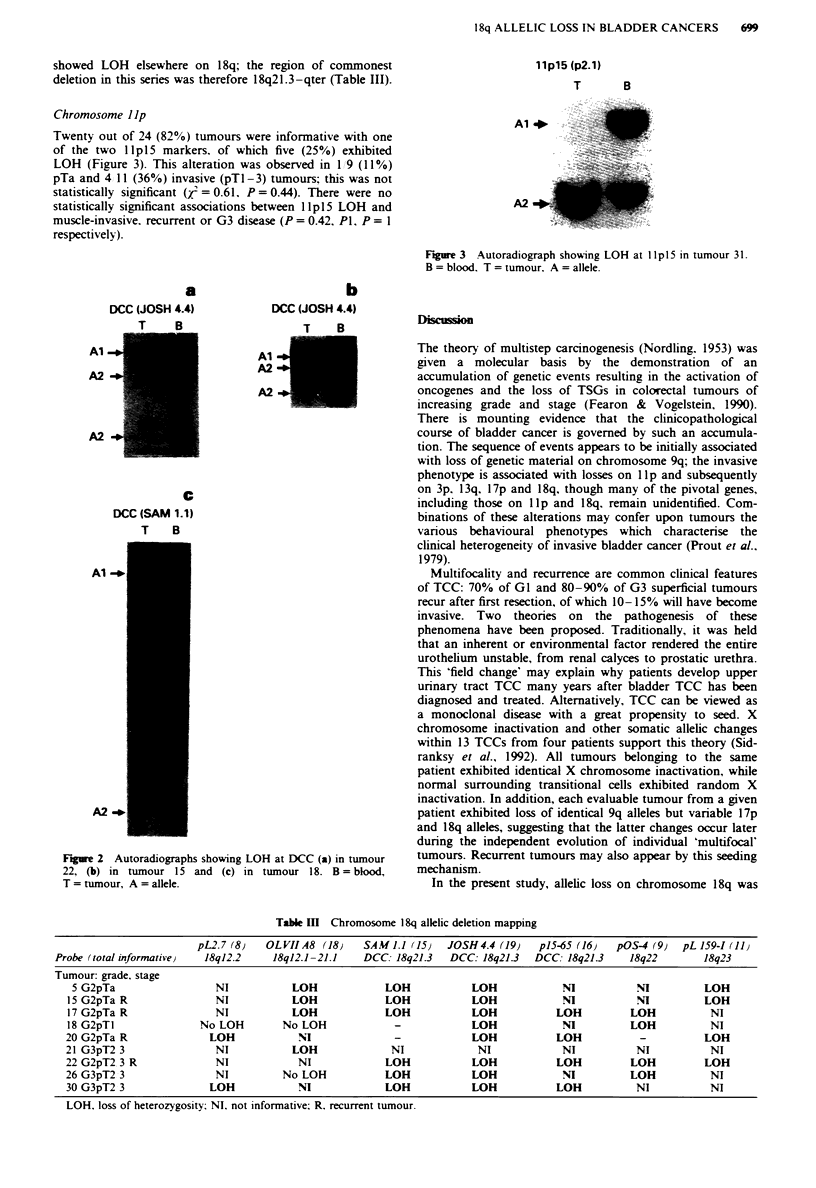

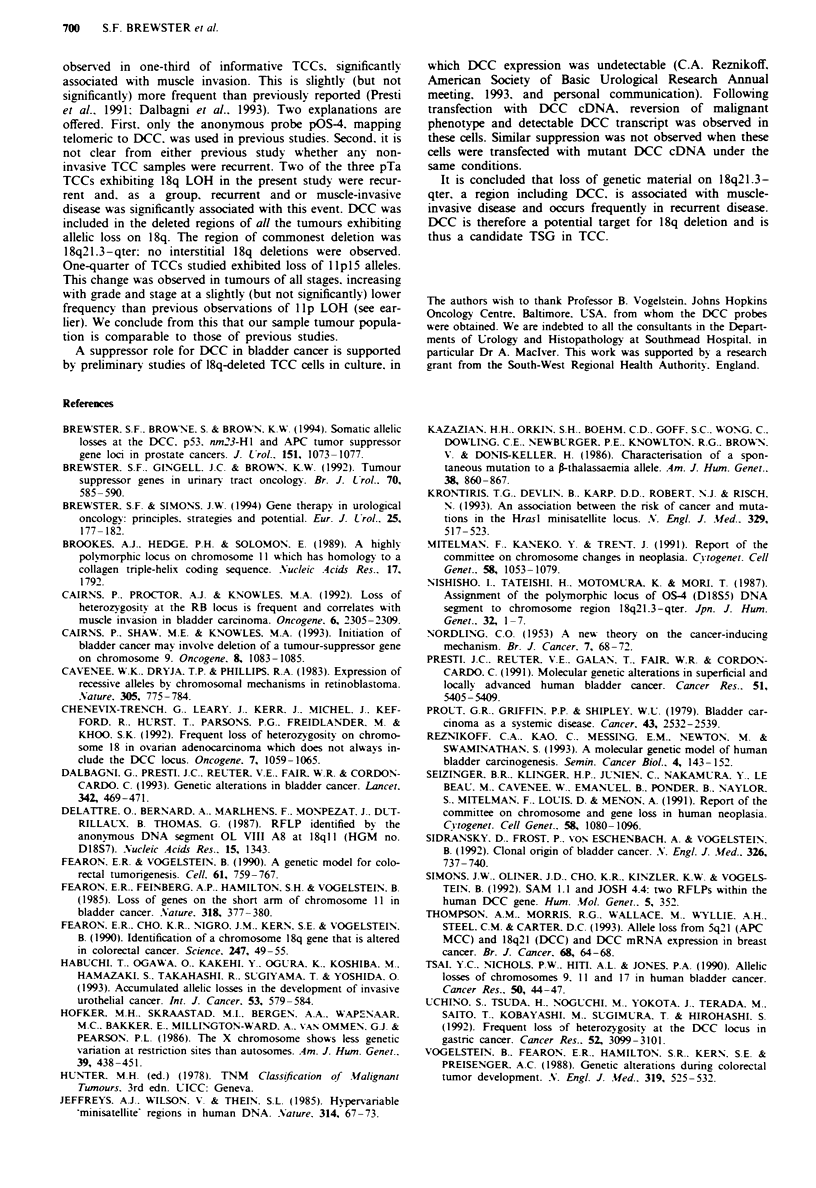


## References

[OCR_00471] Brewster S. F., Browne S., Brown K. W. (1994). Somatic allelic loss at the DCC, APC, nm23-H1 and p53 tumor suppressor gene loci in human prostatic carcinoma.. J Urol.

[OCR_00474] Brewster S. F., Gingell J. C., Brown K. W. (1992). Tumour suppressor genes in urinary tract oncology.. Br J Urol.

[OCR_00479] Brewster S. F., Simons J. W. (1994). Gene therapy in urological oncology: principles, strategies and potential.. Eur Urol.

[OCR_00484] Brookes A. J., Hedge P. H., Solomon E. (1989). A highly polymorphic locus on chromosome 11 which has homology to a collagen triple-helix coding sequence.. Nucleic Acids Res.

[OCR_00490] Cairns P., Proctor A. J., Knowles M. A. (1991). Loss of heterozygosity at the RB locus is frequent and correlates with muscle invasion in bladder carcinoma.. Oncogene.

[OCR_00494] Cairns P., Shaw M. E., Knowles M. A. (1993). Initiation of bladder cancer may involve deletion of a tumour-suppressor gene on chromosome 9.. Oncogene.

[OCR_00499] Cavenee W. K., Dryja T. P., Phillips R. A., Benedict W. F., Godbout R., Gallie B. L., Murphree A. L., Strong L. C., White R. L. Expression of recessive alleles by chromosomal mechanisms in retinoblastoma.. Nature.

[OCR_00507] Chenevix-Trench G., Leary J., Kerr J., Michel J., Kefford R., Hurst T., Parsons P. G., Friedlander M., Khoo S. K. (1992). Frequent loss of heterozygosity on chromosome 18 in ovarian adenocarcinoma which does not always include the DCC locus.. Oncogene.

[OCR_00513] Dalbagni G., Presti J., Reuter V., Fair W. R., Cordon-Cardo C. (1993). Genetic alterations in bladder cancer.. Lancet.

[OCR_00516] Delattre O., Bernard A., Marlhens F., Monpezat J. P., Dutrillaux B., Thomas G. (1987). RFLP identified by the anonymous DNA segment OL VII A8 at 18q11 (HGM8 no. D18S7).. Nucleic Acids Res.

[OCR_00526] Fearon E. R., Feinberg A. P., Hamilton S. H., Vogelstein B. Loss of genes on the short arm of chromosome 11 in bladder cancer.. Nature.

[OCR_00522] Fearon E. R., Vogelstein B. (1990). A genetic model for colorectal tumorigenesis.. Cell.

[OCR_00539] Habuchi T., Ogawa O., Kakehi Y., Ogura K., Koshiba M., Hamazaki S., Takahashi R., Sugiyama T., Yoshida O. (1993). Accumulated allelic losses in the development of invasive urothelial cancer.. Int J Cancer.

[OCR_00546] Hofker M. H., Skraastad M. I., Bergen A. A., Wapenaar M. C., Bakker E., Millington-Ward A., van Ommen G. J., Pearson P. L. (1986). The X chromosome shows less genetic variation at restriction sites than the autosomes.. Am J Hum Genet.

[OCR_00555] Jeffreys A. J., Wilson V., Thein S. L. (1985). Hypervariable 'minisatellite' regions in human DNA.. Nature.

[OCR_00559] Kazazian H. H., Orkin S. H., Boehm C. D., Goff S. C., Wong C., Dowling C. E., Newburger P. E., Knowlton R. G., Brown V., Donis-Keller H. (1986). Characterization of a spontaneous mutation to a beta-thalassemia allele.. Am J Hum Genet.

[OCR_00566] Krontiris T. G., Devlin B., Karp D. D., Robert N. J., Risch N. (1993). An association between the risk of cancer and mutations in the HRAS1 minisatellite locus.. N Engl J Med.

[OCR_00581] NORDLING C. O. (1953). A new theory on cancer-inducing mechanism.. Br J Cancer.

[OCR_00588] Presti J. C., Reuter V. E., Galan T., Fair W. R., Cordon-Cardo C. (1991). Molecular genetic alterations in superficial and locally advanced human bladder cancer.. Cancer Res.

[OCR_00591] Prout G. R., Griffin P. P., Shipley W. U. (1979). Bladder carcinoma as a systemic disease.. Cancer.

[OCR_00597] Reznikoff C. A., Kao C., Messing E. M., Newton M., Swaminathan S. (1993). A molecular genetic model of human bladder carcinogenesis.. Semin Cancer Biol.

[OCR_00607] Sidransky D., Frost P., Von Eschenbach A., Oyasu R., Preisinger A. C., Vogelstein B. (1992). Clonal origin bladder cancer.. N Engl J Med.

[OCR_00612] Simons J. W., Oliner J. D., Cho K. R., Kinzler K. W., Vogelstein B. (1992). SAM 1.1 and JOSH 4.4: two RFLPs within the human DCC gene.. Hum Mol Genet.

[OCR_00617] Thompson A. M., Morris R. G., Wallace M., Wyllie A. H., Steel C. M., Carter D. C. (1993). Allele loss from 5q21 (APC/MCC) and 18q21 (DCC) and DCC mRNA expression in breast cancer.. Br J Cancer.

[OCR_00623] Tsai Y. C., Nichols P. W., Hiti A. L., Williams Z., Skinner D. G., Jones P. A. (1990). Allelic losses of chromosomes 9, 11, and 17 in human bladder cancer.. Cancer Res.

[OCR_00628] Uchino S., Tsuda H., Noguchi M., Yokota J., Terada M., Saito T., Kobayashi M., Sugimura T., Hirohashi S. (1992). Frequent loss of heterozygosity at the DCC locus in gastric cancer.. Cancer Res.

[OCR_00634] Vogelstein B., Fearon E. R., Hamilton S. R., Kern S. E., Preisinger A. C., Leppert M., Nakamura Y., White R., Smits A. M., Bos J. L. (1988). Genetic alterations during colorectal-tumor development.. N Engl J Med.

